# Supplementary material for: Systematic Investigation of the Multifaceted Role of SOX11 in Cancer
Source: Cancers (Basel). 2022 Dec 12;14(24):6103. doi: 10.3390/cancers14246103 (PMC9776339; doi:10.3390/cancers14246103)
Supplement: Supplementary file 1 [file cancers-14-06103-s001.zip › cancers-1964496-supplementary.pdf]

Supplementary materials

Systematic Investigation of the Multifaceted Role of *SOX11* in Cancer

Qingqing Sun, Jun Du, Jie Dong, Shuaikang Pan, Hongwei Jin, Xinghua Han and Jingguo Zhang

Table S1. The protein expression of *SOX11* in pan-cancer by tissue microarray.

Cancer type	*n*	*SOX11* expression		*p*-value
		High	Low	
THCA				1.000
Cancer	2	2	0	
Normal	6	4	2	
ESCA				0.012*
Cancer	3	3	0	
Normal	6	0	6	
STAD				0.083
Cancer	3	1	2	
Normal	6	6	0	
COAD				0.070
Cancer	6	6	0	
Normal	7	3	4	
READ				0.143
Cancer	4	4	0	
Normal	3	1	2	
LIHC				0.182
Cancer	5	5	0	
Normal	6	3	3	
PAAD				0.444
Cancer	5	3	2	
Normal	4	4	0	
LUSC				0.024*
Cancer	4	3	1	
Normal	7	0	7	
LUAD				0.002*
Cancer	4	4	0	
Normal	8	0	8	
BRCA				0.200
Cancer	4	4	0	
Normal	1	0	1	
KIRC				0.018*
Cancer	5	0	5	
Normal	3	3	0	

*, $p < 0.05$.

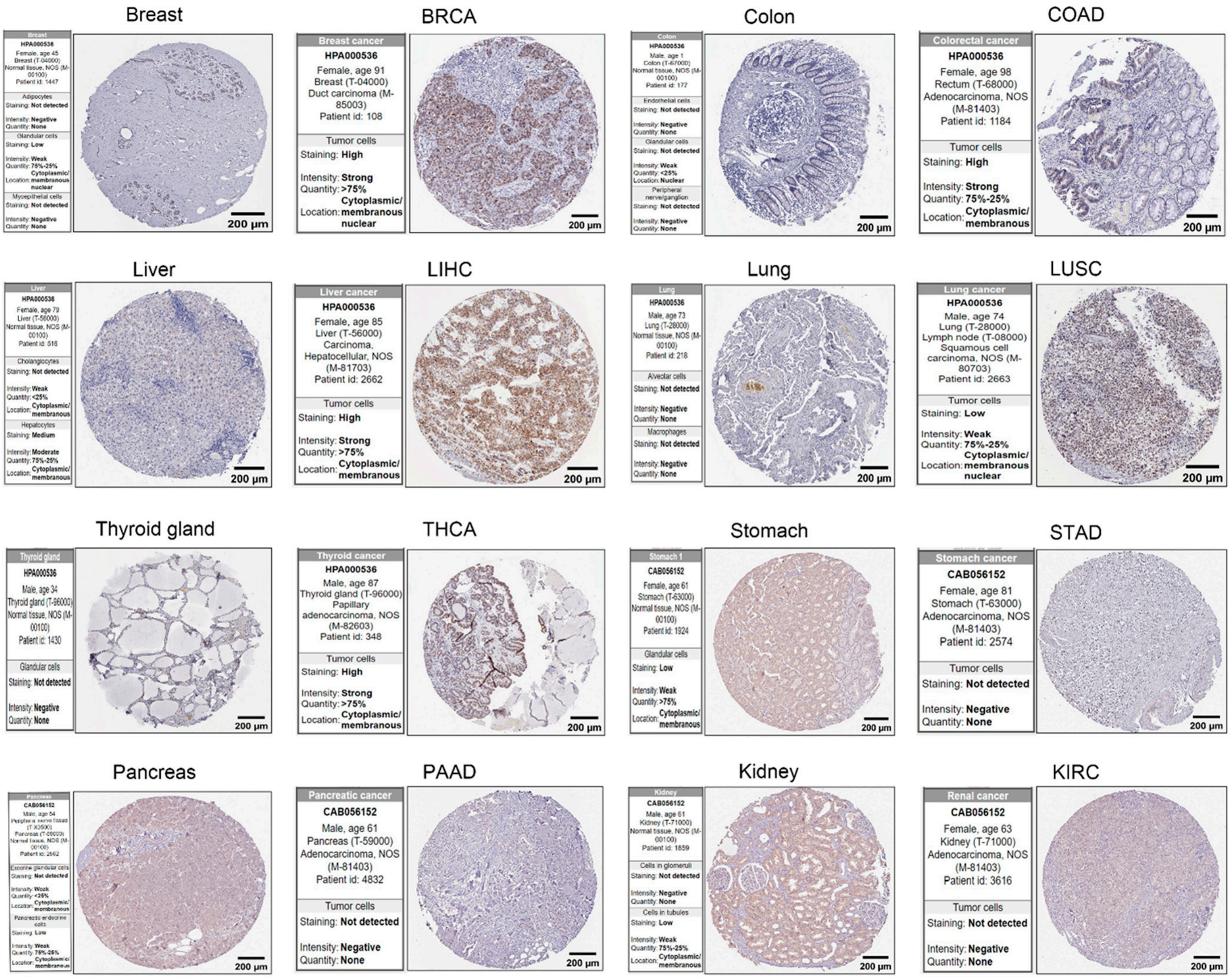

Figure S1. Protein expression level of *SOX11* in cancer and corresponding normal tissues from the HPA da-tabase of BRCA, COAD, LIHC, LUSC, THCA, STAD, PAAD, KIRC. Representative images of *SOX11* expression in pancancer tissues are shown. Original magnification, ×40, Scale bar: black, 200 µm..

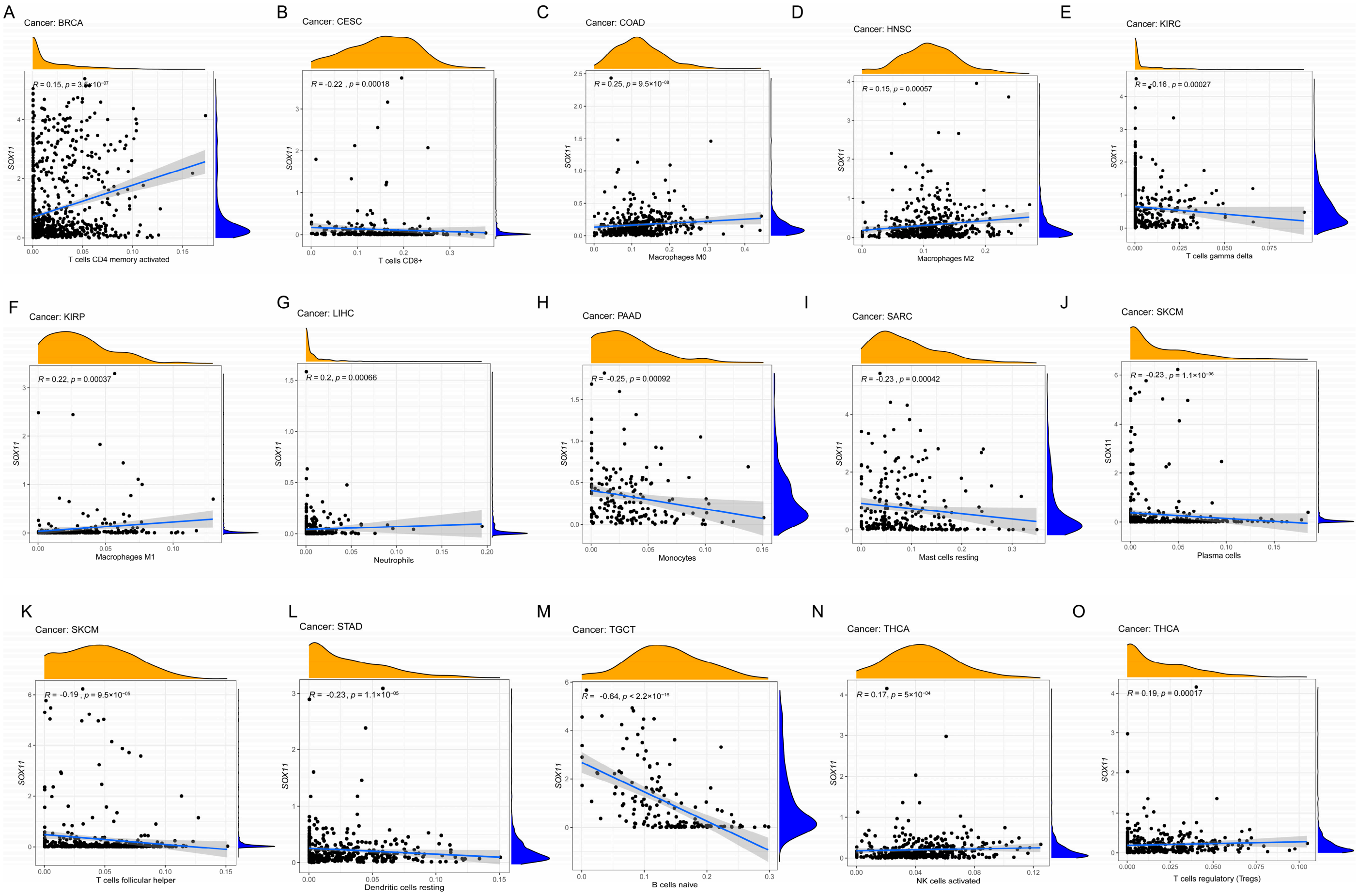

Figure S2. (A) Correlation between CD4+ T cell infiltration and *SOX11* expression in BRCA. (B) Correlation between CD8+ T cell infiltration and *SOX11* expression in CESC. (C) Correlation between macrophage M0 infiltration and *SOX11* expression in COAD. (D) Correlation between macrophage M2 cell infiltration and *SOX11* expression in HNSC. (E) Correlation between gamma delta T cell infiltration and *SOX11* expression in KIRC. (F) Correlation between macrophage M1 cell infiltration and *SOX11* expression in KIRP. (G) Correlation between neutrophil infiltration and *SOX11* expression in LIHC. (H) Correlation between monocyte cell infiltration and *SOX11* expression in PAAD. (I) Correlation between mast cell infiltration and *SOX11* expression in SARC. (J) Correlation between plasma cell infiltration and *SOX11* expression in SKCM. (K) Correlation between T follicular helper cell infiltration and *SOX11* expression in SKCM. (L) Correlation between dendritic cell infiltration and *SOX11* expression in STAD. (M) Correlation between B cell infiltration and *SOX11* expression in TGCT. (N) Correlation between NK cell infiltration and *SOX11* expression in THCA. (O) Correlation between Treg cell infiltration and *SOX11* expression in THCA.

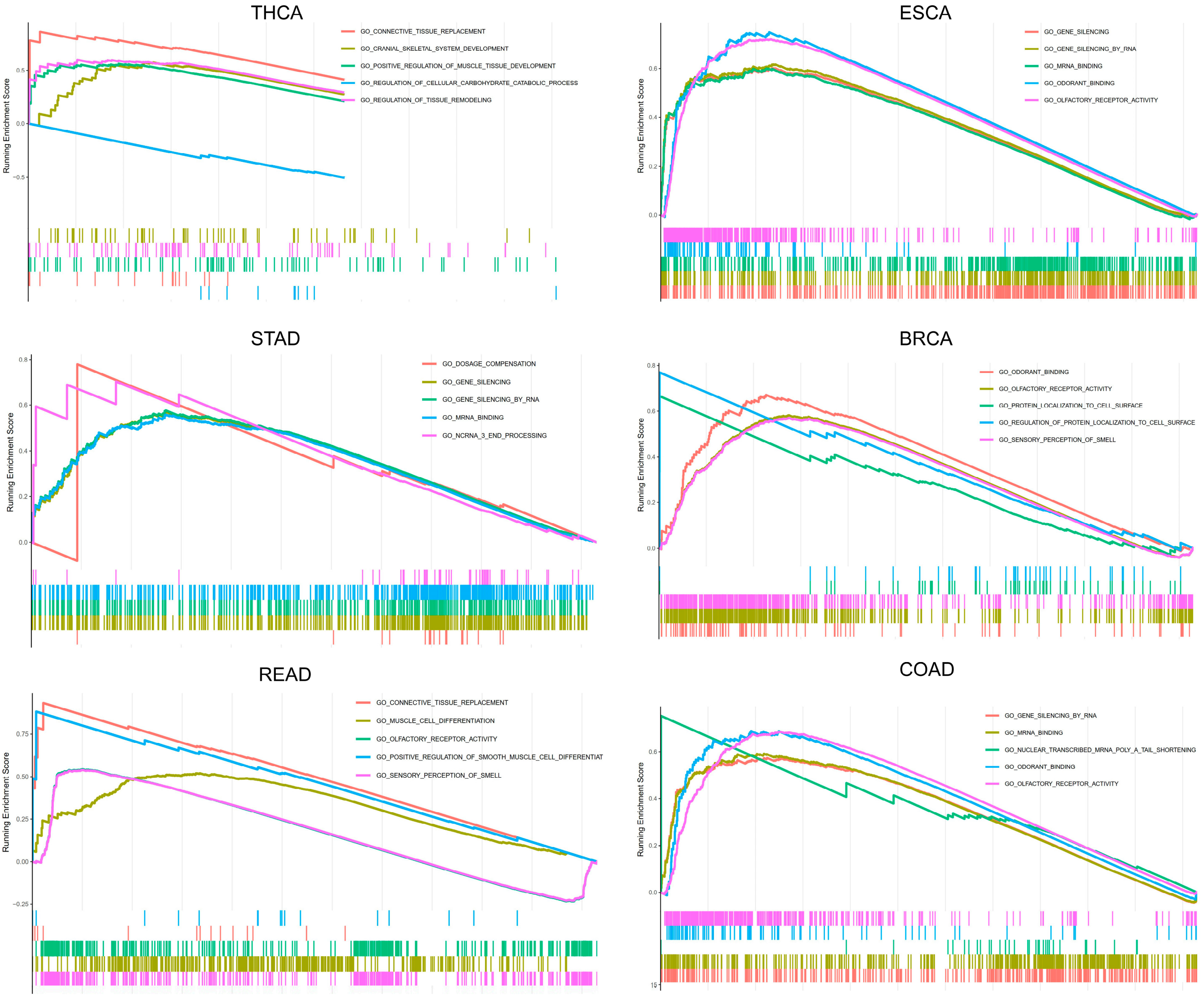

Figure S3. The Gene Set Enrichment Analysis (GSEA) in Gene Ontology (GO) signature of THCA, ESCA, STAD, BRCA, READ and COAD. The curves with different colors indicate the different molecu-lar functions. Peaks in the upward direction indicate positive regulation by *SOX11* and peaks in the downward direction represent negative regulation by *SOX11*. (A) The GSEA analysis of *SOX11*-mediated molecular function in THCA; (B) The GSEA analysis of *SOX11*-mediated mo-lecular function in ESCA; (C) The GSEA analysis of *SOX11*-mediated molecular function in STAD; (D) The GSEA analysis of *SOX11*-mediated molecular function in BRCA; (E) The GSEA analysis of *SOX11*-mediated molecular function in READ; (F) The GSEA analysis of *SOX11*-mediated mo-lec-ular function in COAD.

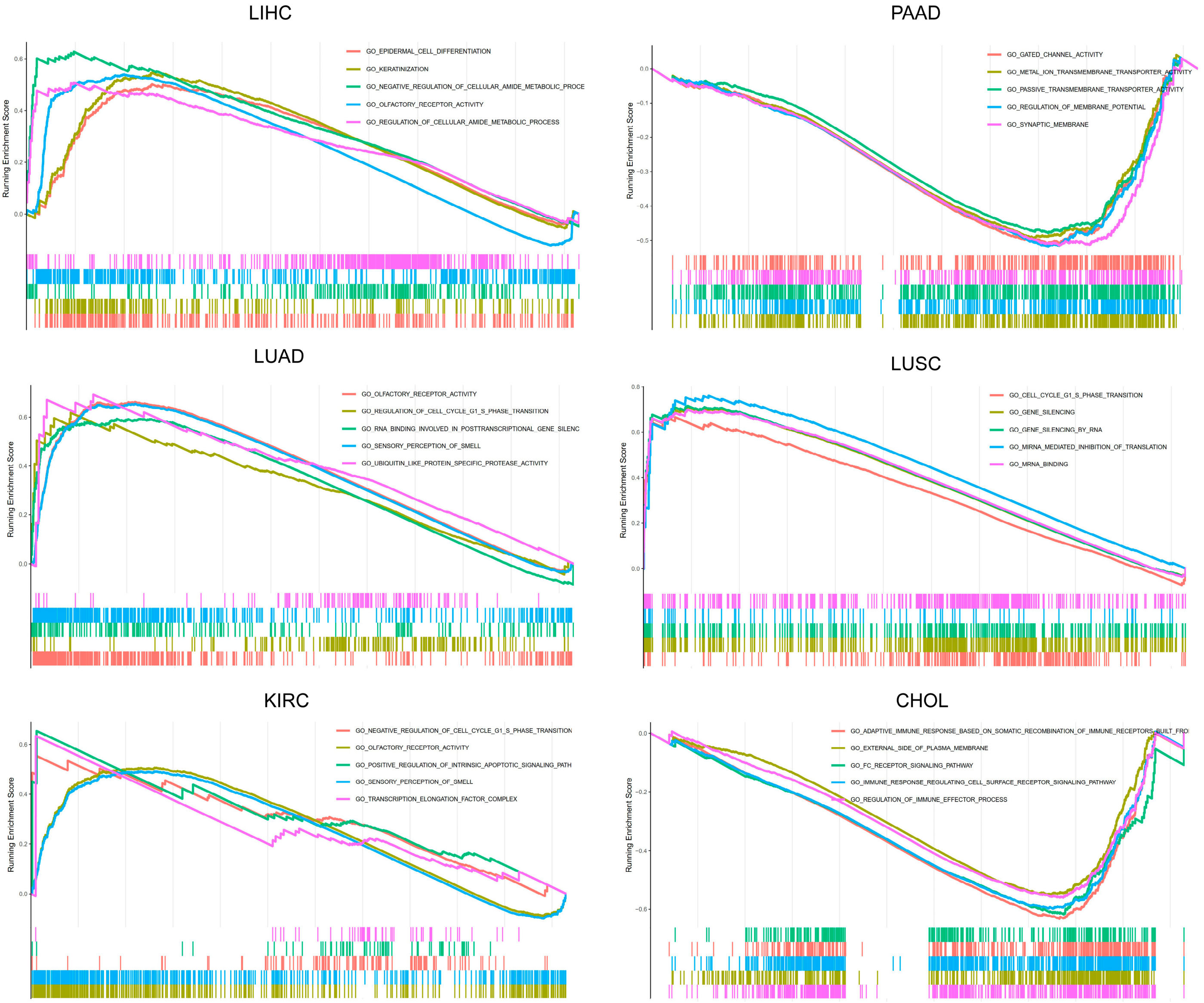

Figure S4. The Gene Set Enrichment Analysis (GSEA) in Gene Ontology (GO) signature of LIHC, PAAD, LUAD, LUSC, KIRC and CHOL. The curves with different colors indicate the different molecular functions. Peaks in the upward direction indicate positive regulation by *SOX11* and peaks in the downward direction represent negative regulation by *SOX11*. (A) The GSEA analysis of *SOX11*-mediated molecular function in LIHC; (B) The GSEA analysis of *SOX11*-mediated molecular function in PAAD; (C) The GSEA analysis of *SOX11*-mediated molecular function in LUAD; (D) The GSEA analysis of *SOX11*-mediated molecular function in LUSC; (E) The GSEA analysis of *SOX11*-mediated molecular function in KIRC; (F) The GSEA analysis of *SOX11*-mediated molecular function in CHOL.
